# Case of Poisoning by Oil of Bitter Almonds

**Published:** 1878-10

**Authors:** 


CASE OF POISONING BY OIL OF BITTER ALMONDS.—On September 14, 1878, I prescribed an emulsion of oil for Mr. H., a man in good flesh, and extraordinary vitality, but who had been suffering with phthisis for the past six months, the apex of the left lung being affected. One-third of the oil emulsion should have been almond-water. By an error of a druggist, four ounces of oil of bitter almonds, instead of four ounces of almond-water, were used in forming the emulsion of twelve ounces. The next day, between 10 and 11 o'clock, Mr. H. took a large tablespoonful of this mixture, having previously taken in the morning a tablespoonful of a cough syrup, containing one drachm of acid, hydrocyan. dil. to eight ounces of menstruum. Soon after taking a dose of the emulsion he felt sick, and took another spoonful of the cough mixture. At 12 M. he felt badly, and went to bed, thinking he was going to have a chill.

I was summoned in haste at 4 P.M. I met Dr. H. at the gate, who said the patient was in a bad condition as the result of my medicine, and that he had given him morphia and bismuth, and ordered milk. I found the patient almost pulseless, extremities cold, lips black, with face intensely cyanosed, looking like a corpse that had lain for hours in the sun. He gasped for breath like a fish out of water, and his speech was unintelligible. I gave him brandy, letting it run from a spoon down his throat, and kept an almost constant stream of cold water pouring on his head. After half an hour his circulation was much improved, and he complained of nausea and vomited what he had eaten the previous day, the result of having been freely dosed with mustard before my arrival. I continued the cold effusions and stimulants till 6.30 P.M.

At 7 P.M. his lips were still livid, but the pulse was good, and the face rapidly regaining its natural appearance. Twelve hours after I found him sitting up, complaining only of numbness of the extremities and weakness.

The principal subjective symptom in this case was intense pain in the head. As he recovered, he continued exclaiming, in a jerking, hesatating way, "What's the mat-matter with me?" I judge, from the size of the spoon, that nearly or quite two drachms of the oil were taken.—*Louisville American Practitioner.*

---

A LARGE HEART.—Dr. John Morris, of Baltimore, writes us: "Mr. John H. Weaver, a well-known undertaker of this city, died in May, 1877, after an illness of nearly two years. His disease was diagnosed, during life, as hypertrophy of the heart, with valvular insufficiency. Examination of the heart, after death, revealed the following conditions: Distance from apex of left ventricle to the origin of aorta, six and one-third inches; thickness of left ventricle one and one-third inches; right ventricle one-third of an inch; weight *forty-four ounces.* It may be added that the valve held water very imperfectly."—*Medical and Surgical Reporter.*

